# Can secretory immunoglobulin A in saliva predict a change in lung infection status in patients with cystic fibrosis? A prospective pilot study

**DOI:** 10.1002/hsr2.52

**Published:** 2018-07-12

**Authors:** Mikkel Christian Alanin, Tania Pressler, Kasper Aanaes, Claus Thorn Ekstrøm, Marianne Skov, Helle Krogh Johansen, Kim G. Nielsen, Christian von Buchwald, Niels Høiby

**Affiliations:** ^1^ Department of Otorhinolaryngology, Head and Neck Surgery and Audiology Rigshospitalet Denmark; ^2^ Copenhagen CF Centre Rigshospitalet Denmark; ^3^ Section of Biostatistics University of Copenhagen Denmark; ^4^ Department of Clinical Microbiology Rigshospitalet Denmark; ^5^ Paediatric Pulmonary Service Rigshospitalet, University of Copenhagen Denmark; ^6^ Institute of Immunology and Microbiology, University of Copenhagen Denmark

**Keywords:** IgA antibodies, Pseudomonas, sinusitis

## Abstract

**Background:**

Chronic lung infection with *Pseudomonas aeruginosa* is the main cause of mortality in patients with cystic fibrosis (CF). Sinus colonization with P. aeruginosa often precedes intermittent lung colonization, and intermittent colonization precedes chronic infection.When P. aeruginosa colonizes the sinuses, elevated immunoglobulin A (IgA) levels specific against P. aeruginosa can be detected in saliva. Therefore, we hypothesized that increasing levels of IgA in saliva can be detected before P. aeruginosa lung colonization.

**Methods:**

Forty‐nine CF patients free from lung colonization with P. aeruginosa or other Gram‐negative bacteria (GNB) were included in this prospective study. Saliva and serum samples were collected and examined for IgA antibodies against P. aeruginosa with at least 6‐month intervals between sequential samples.

**Results:**

A total of 110 measurements of IgA in saliva were included. During a median of 8.5‐month follow‐up, 25 patients changed their lung infection status. We were able to construct a statistical model that for a given value of IgA in saliva, could predict the probability of a change in lung infection status within the next 8.5 months (median): *p* = 1 / (1 + exp(−(−0.9582 + 1.6518*IgA)). The model includes a prediction band where 95% of new measurements are predicted to fall within. The model, however, failed to reach statistical significance (*P* = 0.056 1‐tailed), probably because of lack of power.

**Conclusion:**

The saliva IgA model may predict a worsening in lung infection status presumably acting as a surrogate marker of P. aeruginosa bacterial sinusitis. The model may identify patients at risk of subsequent lung colonization and, thus, be a helpful clinical tool, but it should be tested in studies with larger sample sizes to evaluate its utility.

## Abstract 2

Background

Chronic lung infection with *Pseudomonas aeruginosa* is the main cause of mortality in patients with cystic fibrosis (CF). Sinus colonization with P. aeruginosa often precedes intermittent lung colonization, and intermittent colonization precedes chronic infection.When P. aeruginosa colonizes the sinuses, elevated immunoglobulin A (IgA) levels specific against P. aeruginosa can be detected in saliva. Therefore, we hypothesized that increasing levels of IgA in saliva can be detected before P. aeruginosa lung colonization.

Methods

Forty‐nine CF patients free from lung colonization with P. aeruginosa or other Gram‐negative bacteria (GNB) were included in this prospective study. Saliva and serum samples were collected and examined for IgA antibodies against P. aeruginosa with at least 6‐month intervals between sequential samples.

Results

A total of 110 measurements of IgA in saliva were included. During a median of 8.5‐month follow‐up, 25 patients changed their lung infection status. We were able to construct a statistical model that for a given value of IgA in saliva, could predict the probability of a change in lung infection status within the next 8.5 months (median): *p* = 1 / (1 + exp(−(−0.9582 + 1.6518*IgA)). The model includes a prediction band where 95% of new measurements are predicted to fall within. The model, however, failed to reach statistical significance (*P* = 0.056 1‐tailed), probably because of lack of power.

Conclusion

The saliva IgA model may predict a worsening in lung infection status presumably acting as a surrogate marker of P. aeruginosa bacterial sinusitis. The model may identify patients at risk of subsequent lung colonization and, thus, be a helpful clinical tool, but it should be tested in studies with larger sample sizes to evaluate its utility.

## 1 INTRODUCTION

Chronic lung infection with *Pseudomonas aeruginosa* is associated with bronchopulmonary inflammation, tissue damage, bronchiectasis, and progressive declining lung function, and is the main cause of premature death or lung transplantation in patients with cystic fibrosis (CF).^1^ Intermittent lung colonization precedes chronic infection,^2^ and preventing or postponing chronicity is of paramount importance in CF treatment.^3^ Important indicators for chronic lung infection include increasing levels of Immunoglobulin G (IgG) antibodies against P. aeruginosa in serum 2 to 3 years before the lung infections becomes chronic and growth of mucoid P. aeruginosa strains in sputum.^4^ Respiratory infection with P. aeruginosa causes increases of antibodies against P. aeruginosa both systemically^5^ and locally in saliva, tears, and nasal secretions.^6^ Secretory immunoglobulin A (s‐IgA) is the predominating antibody on mucosal surfaces.^6^, ^7^

In the initial stages of lung colonization, P. aeruginosa can usually be eradicated. However, patients are usually re‐colonized with the same clone from the patients' own sinuses; thus, the sinuses can function as a sustainable bacterial reservoir.^8^, ^9^, ^10^ In many cases, colonization of paranasal sinuses may precede intermittent lung colonization.^11^ This encourages efficient treatments of sinuses that may subsequently spare colonization of the lower airways. Unfortunately, no non‐invasive method can currently detect P. aeruginosa sinusitis with a high specificity or sensitivity. Possible methods include nasal lavage^9^ or swabs from the middle meatus; however, in our experience (unpublished), there is a risk of false negative results, because the pathogenic bacteria can be present in, for example, the frontal or sphenoid sinuses. In a diseased sinus with mucosal oedema, nasal lavage may not enter these cavities, encouraging research in other modalities. However, the way of drainage from any paranasal sinus is through its ostium. Therefore, secretions may not be ultimately hidden from sampling, but they will appear in descending airways at some time.

Our research group has shown that when P. aeruginosa colonizes the sinuses, elevated secretory (s) ‐IgA levels can be detected in nasal mucosa and saliva. Further, values are significantly higher in patients' lung colonized with P. aeruginosa or other GNB.^6^

Therefore, we hypothesized that increasing levels of s‐IgA in saliva against P. aeruginosa standard antigen (St‐Ag) precede intermittent P. aeruginosa lung colonization.

In this prospective study, saliva samples were collected and analyzed in order to test if P. aeruginosa or GNB lung colonization could be predicted. If so, we provide a diagnostic antibody assay for early detection of P. aeruginosa sinus colonization, and an eradication attempt could be initiated. In this way, the lungs may be spared from colonization, inflammation, and irreversible lung damage. Eradication regimens include endoscopic sinus surgery (ESS) with adjuvant therapy including systemic antibiotics, nasal irrigation with saline and antibiotics,^12^, ^13^ or nasal inhalation of antibiotics.^14^

## 2 MATERIAL AND METHODS

### 2.1 Patients

The CF diagnosis was based on characteristic clinical features, abnormal sweat electrolytes, and genotypes. All CF patients from the Copenhagen CF Centre are followed in the out‐patient clinic every month. A clinical exam is followed by routine microbiological surveillance of sputum samples or samples obtained by endolaryngeal suction. Lower airway origin of the samples is verified by microscopy. Further, regularly blood samples are taken and analyzed for anti‐bacterial antibodies, as described previously.^6^, ^15^ All patients diagnosed with CF and free from GNB lung infection and colonization followed at the Copenhagen CF Centre from November 1, 2013 to June 1, 2015, were eligible for the study.

### 2.2 Lung infection status

We divided patients into 4 groups according to the bacterial flora^6^, ^15^:

1. Chronically infected with P. aeruginosa (CF + P (c)) was defined as presence of this bacterium in 6 consecutive monthly samples or a shorter period if there were 2 or more precipitating antibodies in serum.
2. Intermittent lung colonization (CF + P (i)) was defined when P. aeruginosa, at monthly visits to the clinic, was isolated occasionally but was *not* present in sputum for 6 months consecutively, and the anti‐pseudomonas IgG antibodies were not elevated but within the normal range (0‐1 precipitating antibodies).
3. If the monthly samples had never contained P. aeruginosa previously, patients were classified as free of P. aeruginosa (CF‐P)
4. Patients colonized/infected in the lungs with other GNB (*Stenotrophomonas maltophilia, Achromobacter xylosoxidans*, or *Burkholderia cepacia complex*) (CF + GNB). These patients were *not* colonized with P. aeruginosa.

### 2.3 Collection of serum and saliva

Saliva samples were collected from all patients with at least 6 months interval between sequential samples from each patient. The blood and saliva samples were obtained simultaneously, as described previously.^6^ In brief, mixed saliva was collected by using 4 sterile 6‐mm diameter filterpaper discs (Whatman AA DISCS 6 mm Cat. No. 2017‐006; Whatman International Ltd, Maidstone, England) which were placed on the oral mucosa for 30 seconds with a forceps or just dipped in a container containing mixed saliva from the patient for at least 30 seconds, as described previously.^16^

### 2.4 IgA and IgG against P. aeruginosa

Eluates of saliva from the paper discs were examined for sIgA, and serum for IgG antibodies, against P. aeruginosa *sonicate* ((St‐Ag) (O serogroups 1‐17)), using enzyme‐linked immunosorbent assays (ELISA), and expressed in optical density values (OD), as reported previously .^6^, ^7^, ^16^ The reason for including serum IgG is, that rise of IgG antibodies is a prognostic sign of a later switch from intermittent to chronic P. aeruginosa lung infection.^4^

### 2.5 Statistics

We investigated whether antibody measurements could predict if a patient would change infection status from non‐colonized to intermittent or chronic infection status, using a generalized linear mixed‐effect logistic regression model. The data span 2 periods, so some patients are available and contribute to 2 sets of before‐after status changes which is why we included a random intercept of subject to account for repeated measurements on some patients. Because of the small number of events, ie, individuals changing status, we were only able to include a single predictor at a time.

The number of observations and events were limited in the dataset, so the logistic regression model could only accommodate a single predictor before running into problems with complete separation. Consequently, we first examined if there was any effect of period, because the 2 periods span different lengths, but no difference was found. We examined each of the 2 predictors independently using a logistic regression model with an initial model that included a (log) linear effect of the predictor as well as the predictor squared to allow for a nonlinear relationship between the log odds of changing status and the predictor. However, allowing for nonlinearity did not contribute anything statistically significant to the model for any of the predictors, and, therefore, we report the results from the logistic regression model.

Data were analysed using SAS (SAS Institute Inc., version 9.4, NC, USA).

### 2.6 Ethics

The study was approved by the local ethics committee (H‐1‐2013‐032). Informed consent was obtained from all patients. Parental consent was obtained for patients <18 years of age. Obtaining the saliva samples did not cause any discomfort. This study was carried out in accordance with the principles of the Helsinki Declaration.

## 3 RESULTS

Forty‐nine CF patients (28/21 female/male, median age 15 years, range 5‐45 years) free from lung colonization with P. aeruginosa (CF‐P) or other GNB, were included in this prospective study. Thirty‐two patients were dF508 homozygous, 15 were dF508 heterozygous, and 2 had other mutations.

**Figure 1:**
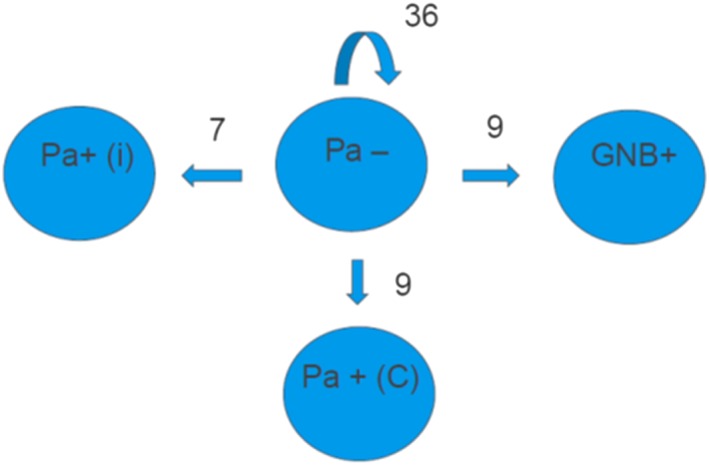
Change in lung infection status. Forty‐nine CF patients free from GNB lung colonization were included in this prospective study. Repeated saliva samples were obtained from each of the patients with at least a 6‐month interval. However, 21 samples from our prospective study in 2009^6^ were also included, to increase power. Consequently, 110 measurements from 49 patients were included. In the 49 patients, we observed 61 events where patients could change in lung infection status. The numbers in Table 1 refer to these events. During a median of 8.5‐months follow‐up, 7 patients changed to intermittent lung colonization with *P. aeruginosa* (Pa + (i)), 9 patients changed to chronic lung infection with *P. aeruginosa* (Pa + (c)) and 9 patients changes status to lung colonization with other gram‐negative bacteria (GNB+)

A total of 110 measurements of s‐IgA in saliva against St‐Ag and IgG in serum against St‐Ag were analyzed from 49 CF patients. To increase power, 21 measurements (from 21 patients) obtained in our prospective study in 2009^6^ were included in these 110 measurements. We only included measurements on patients who had already agreed to participate in the present study. During a median of 8.5 months follow‐up (range 6‐66 months), 25 patients changed their infection status. Seven patients changed from free of lung colonization to intermittent lung colonization, 9 changed to chronic lung infection, and 9 patients changed to lung colonization or infection with other GNB (Figure 1).

### 3.1 s‐IgA

Mean s‐IgA was higher in patients who changed lung infection status compared with patients who remained free from lung colonization: the mean OD was 0.50 and 0.33, respectively (Table 1).

**Table 1:** Saliva s‐IgA (OD) against P. aeruginosa St‐Ag in CF patients who remained free of colonization/infection or who became colonized or infected with P. aeruginosa or other gram‐negative bacteria^a^. LowerQ, upper Q = 25% and 75% quantiles, respectively

Patients' Final Lung Status	Number of Observations	Median sIgA	LowerQ sIgA	UpperQ sIgA
Remained free of colonization/infection	36	0.24	0.21	0.38
Became colonized/infected	25	0.38	0.23	0.55

a *Burkholderia species, Achromobacter xylosoxidans, Stenotrophomonas maltophilia*.

Based on the s‐IgA measurements in patients who remained free of colonization or infection compared with patients with a change in lung infection status, we constructed a statistical model such that for a given value of s‐IgA against St‐Ag in saliva, it can predict the probability of a change (worsening) in lung infection status within the next 8.5 months (median): *p* = 1 / (1 + exp(−(−0.9582 + 1.6518*IgA)).

**Figure 2:**
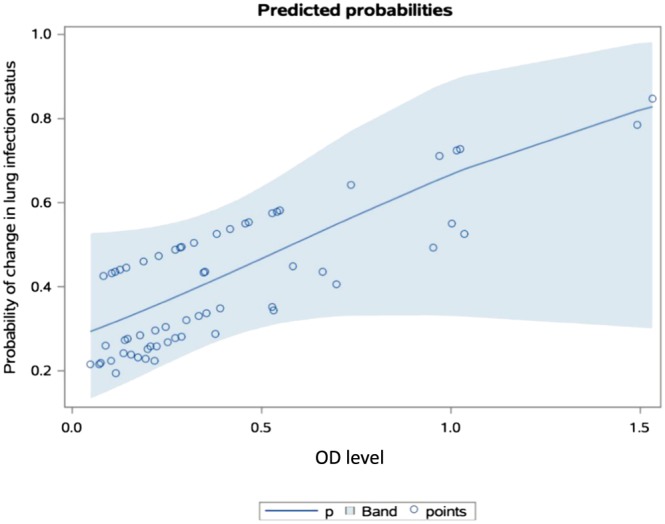
Predicted probability of a change in lung infection status for a given s‐IgA saliva measurement. This model predicts a probability for a change in lung infection status for a given IgA OD value, *P* = 1 / (1 + exp(−(−0.9582 + 1.6518* within. IgA)). The prediction band indicates where 95% of new measurements are predicted to fall. The points in the figure show the predicted probabilities. Y axis is the probability of a change in lung infection status. X axis refers to the IgA level in saliva

The model is plotted in Figure 2 and includes a prediction band where 95% of new measurements are predicted to fall within. The model, however, failed to reach statistical significance, as the probability was calculated to be *P* = 0.056, 1‐tailed, using a likelihood‐ratio test.

### 3.2 IgG

Mean serum IgG was higher in patients who changed their lung infection status compared with patients who remained free from lung colonization (Table 2). However, the model was inferior compared with the one with s‐IgA, with regard to detecting a change (*P* = 0.16, 1‐ tailed), using a likelihood‐ratio test.

**Table 2:** Serum IgG (OD) against P. aeruginosa St‐Ag in CF patients who remained free of colonization/infection or who became colonized or infected with P. aeruginosa or other gram‐negative bacteria^a^. LowerQ, upper Q = 25% and 75% quantiles, respectively

Patients' Final Lung Status	Number of Observations	Median IgG	LowerQ IgG	UpperQ IgG
Remained free of colonization/infection	43	1.61	1.06	2.19
Became colonized/infected	25	2.00	0.23	2.50

a *Burkholderia species, Achromobacter xylosoxidans, Stenotrophomonas maltophilia*.

## 4 DISCUSSION

We have previously reported that patients' lung colonized with P. aeruginosa or other GNB have higher levels of s‐IgA against P. aeruginosa St‐Ag in saliva than patients free of lung colonization.^6^ This may result from a local mucosal antibody response to P. aeruginosa when it first colonizes the sinuses.

Sinus colonization often precedes intermittent lung colonization with P. aeruginosa, 11 and intermittent colonization precedes chronic lung infection.^2^

Most interestingly, our data provides preliminary evidence that our saliva s‐IgA model may predict an early worsening in lung infection status, presumably acting as a surrogate marker of P. aeruginosa or other GNB mediated bacterial sinusitis and, thereby, identifying patients at risk of subsequent lung colonization and infection with GNB. However, the model did not reach statistical significance probably due to limited power, and it must, therefore, be confirmed in larger prospective studies. Nevertheless, the results from this study are supported by a recent study by Mauch et al^17^ who concluded that s‐IgA measurement can be used as a screening model for patients at risk of chronic infection.

Measuring IgG levels in serum is a useful tool for identifying patients at risk of becoming chronically lung infected,^4^ and we showed that the s‐IgA model was superior to serum IgG in detecting early colonization. In this way, the 2 assays may very well supplement each other consecutively, and we recommend that specific antibodies in both saliva and serum should be measured regularly.

The reason why s‐IgA is superior at early infections may be a result of the different action of the immune system in early versus late infections. When the sinuses are colonized by P. aeruginosa, the immune response is dominated by a high production of sIgA that binds to P. aeruginosa antigens and prevent complement activation and the recruitment of polymorphonuclear leucocytes, thereby, reducing the inflammatory and systemic response.^8^, ^18^

Both ESS with adjuvant therapy^12^, ^13^ and nasal inhalation of antibiotics^14^ can eradicate sinus bacteria, highlighting the importance of timely intervention against a potential sinonasal bacterial reservoir.

Whether a given s‐IgA value may be helpful to identify patients who should be offered a sinus eradication attempt even before intermittent lung colonization occurs, should be investigated in a clinical trial.

Both ESS^13^ and administration of topical nasal antibiotics^19^ are safe procedures in CF.

Our study has other limitations. The s‐IgA model cannot reliably distinguish between lung colonization with P. aeruginosa or other GNB due to cross‐reaction of antibodies against common antigens in these bacteria, unless absorption studies are carried out.^20^ However, ESS has also proven successful in eradicating GNB other than P. aeruginosa, 13 minimizing the importance of exact identification of the pathogen by serological methods. Nevertheless, there certainly are different strategies for how aggressively to treat different GNB.

Another limitation of this study is the usage of s‐IgA measurements from our previous published paper.^6^

Pending on better modalities for detecting upper airway *P. aeruginosa* colonization, we believe that s‐IgA saliva measurements may be a helpful clinical tool. Obtaining saliva samples is very easy and without any discomfort to the patients including children, and the IgG assay is commercially available and can easily be modified for detection of sIgA.^11^

## FUNDING

The Novo Nordisk Foundation supported H.K.J. as a clinical research stipend. K.G.N. received funding from the European Union Seventh Framework Programme (FP7/2007‐2013) under grant agreement n8305404 (BESTCILIA).

## CONFLICTS OF INTEREST

None declared.

## AUTHOR CONTRIBUTIONS

Conceptualization: Mikkel Christian Alanin, Tania Pressler, Kasper Aanaes, Claus Thorn Ekstrøm, Marianne Skov, Helle Krogh Johansen, Kim G. Nielsen, Christian von Buchwald, Niels Høiby

Methodology: Mikkel Alanin, Claus Thorn Eskstrøm, Niels Høiby

Formal analysis: Mikkel Alanin, Claus Thorn Ekstrøm, Niels Høiby

Resources: Niels Høiby

Writing – original draft preparation: Mikkel Christian Alanin

Writing – review and editing: Mikkel Christian Alanin, Tania Pressler, Kasper Aanaes, Claus Thorn Ekstrøm, Marianne Skov, Helle Krogh Johansen, Kim G. Nielsen, Christian von Buchwald, Niels Høiby
